# Evaluation of Antioxidant Capacity and Gut Microbiota Modulatory Effects of Different Kinds of Berries

**DOI:** 10.3390/antiox11051020

**Published:** 2022-05-22

**Authors:** Jiebiao Chen, Yichen Shu, Yanhong Chen, Zhiwei Ge, Changfeng Zhang, Jinping Cao, Xian Li, Yue Wang, Chongde Sun

**Affiliations:** 1 Laboratory of Fruit Quality Biology/Zhejiang Provincial Key Laboratory of Horticultural Plant Integrative Biology/The State Agriculture Ministry Laboratory of Horticultural Plant Growth, Development and Quality Improvement, Zhejiang University, Zijingang Campus, Hangzhou 310058, China; jiebiaochen@zju.edu.cn (J.C.); 3160100268@zju.edu.cn (Y.S.); 0017165@zju.edu.cn (J.C.); xianli@zju.edu.cn (X.L.); adesun2006@zju.edu.cn (C.S.); 2 Laboratory Animal Center of Zhejiang University, Zijingang Campus, Hangzhou 310058, China; chenyanhong@zju.edu.cn; 3 Analysis Center of Agrobiology and Environmental Sciences, Zhejiang University, Zijingang Campus, Hangzhou 310058, China; gezw@zju.edu.cn; 4 Shandong Key Laboratory of Storage and Transportation Technology of Agricultural Products, Shandong Institute of Commerce and Technology, Jinan 250103, China; yuewa@zju.edu.cn; 5 National Engineering Research Center for Agricultural Products Logistics, Jinan 250103, China

**Keywords:** berry, anthocyanin, antioxidant, gut microbiota, short-chain fatty acids

## Abstract

Berries are fairly favored by consumers. Phenolic compounds are the major phytochemicals in berries, among which anthocyanins are one of the most studied. Phenolic compounds are reported to have prebiotic-like effects. In the present study, we identified the anthocyanin profiles, evaluated and compared the antioxidant capacities and gut microbiota modulatory effects of nine common berries, namely blackberry, black goji berry, blueberry, mulberry, red Chinese bayberry, raspberry, red goji berry, strawberry and white Chinese bayberry. Anthocyanin profiles were identified by UPLC-Triple-TOF/MS. In vitro antioxidant capacity was evaluated by four chemical assays (DPPH, ABTS, FRAP and ORAC). In vivo antioxidant capacity and gut microbiota modulatory effects evaluation was carried out by treating healthy mice with different berry extracts for two weeks. The results show that most berries could improve internal antioxidant status, reflected by elevated serum or colonic T-AOC, GSH, T-SOD, CAT, and GSH-PX levels, as well as decreased MDA content. All berries significantly altered the gut microbiota composition. The modulatory effects of the berries were much the same, namely by the enrichment of beneficial SCFAs-producing bacteria and the inhibition of potentially harmful bacteria. Our study shed light on the gut microbiota modulatory effect of different berries and may offer consumers useful consumption guidance.

## 1. Introduction

Epidemiological studies have demonstrated that an increased consumption of fruit and vegetables is inversely correlated with the risk of developing chronic diseases [1]. The health-promoting effects of fruits and vegetables are thought to be attributed to the rich nutrients they contain, such as fiber, essential vitamins and minerals, while mounting shreds of evidence indicate that phytochemicals such as polyphenols and carotenoids may also confer health benefits [2].

Among fruits, berries are fairly favored by consumers because of their attractive colors, luscious taste and high nutritive value. Typically, berries are small, pulpy, juicy, brightly colored and often edible fruits. The most consumed berry types include those from the genera *Rubus* (blackberries, raspberries); *Vaccinium* (blueberries, cranberries); *Fragaria* (strawberries); *Ribes* (black currant); *Lycium* (goji berries); *Myrica* (bayberries); or *Morus* (mulberries) [3,4]. Berries are a rich source of a variety of nutritive compounds such as sugars, essential oils, vitamins and minerals, as well as non-nutritive phytochemicals [5]. Phenolic compounds are the major phytochemicals in berry fruits [4]. They represent a diverse group of compounds including phenolic acids (hydroxybenzoic and hydroxycinnamic acid conjugates), flavonoids (anthocyanins, flavonols, flavones, flavanols, flavanones and isoflavonoids), tannins (proanthocyanidins), stilbenes, and lignins [2,6,7], among which anthocyanins are the most studied. Anthocyanins are natural water-soluble pigments, conferring to plant organs a diversity of colors from orange and red to blue and purple, and they are structurally based on the polyhydroxy or polymethoxy derivatives of 2-phenylbenzopyrylium (flavylium ion). Anthocyanins are present in nature as glycosides. More than 700 anthocyanins have already been identified in plants, and most of them are based on six aglycones (also called anthocyanidins): cyanidin, delphinidin, malvidin, pelargonidin, peonidin, and petunidin [6,7]. Anthocyanins are especially abundant in berries and their profile is mainly determined by berry species and is particularly diversified [3].

Phenolic compounds in berries endow them with various health benefits, such as antioxidant, anti-inflammatory, antihypertensive, antihyperglycemic [8], antimicrobial, antimutagenic and neuroprotective properties [5], and these effects have mostly been attributed to their antioxidant properties [4]. Oxidative stress is triggered by the imbalance between the production of reactive species (ROS) and internal antioxidant defense which is implicated in aging and in the pathology of many chronic diseases [4]. Phenolics in fruits and vegetables are effective dietary antioxidants. Structurally, they tend to donate an electron or a hydrogen atom to a free radical and convert it into an inoffensive molecule [9]. As representative phenolics in berries, anthocyanins act as well-recognized ROS scavengers. They can inactivate ROS directly or indirectly and their antioxidant capacities are even superior to synthetic antioxidants such as Trolox [7].

The bioavailability of phenolic compounds is directly influenced by their chemical structures. Most phenolic compounds (such as anthocyanins) have a low absorption capability in the upper gastrointestinal (GI) tract and reach the colon either intact or in the form of their metabolites. These compounds interact with the gut microbiota reciprocally (that is, the bacterial community help to metabolize these unabsorbed compounds to better exert their bioactivities, while these compounds or corresponding metabolites in turn modulate the composition and functions of microbiota) [10]. Gut microbiota refers to the community of microorganisms living in the GI tract in animals and humans [11] which play a vital role in many aspects of human health, including immune, metabolic and neurobehavioral traits [12].Gut microbiota dysbiosis is closely related to a range of physiological disorders, including low-grade inflammation and metabolic disorders [13]. Diet plays an essential role in modulating the composition and function of the gut microbiota [14], both long- and short-term. Dietary fiber and polyphenols that are present in fruit and vegetables can be utilized by the gut microbiota to produce health-promoting short-chain fatty acids (SCFAs) and other small molecules [15]. Thus, polyphenols may manage chronic diseases through a gut microbiota-mediated manner.

Berries are good candidates to study the mutual relationship between phenolics and gut microbiota due to their rich and diverse phenolic profiles [16]. However, few studies have compared the gut microbiota modulatory effects of different types of berries on healthy objects. Therefore, the main goal of the present study was to evaluate and compare the in vitro and in vivo antioxidant capacities, anthocyanin profiles and gut microbiota modulatory effects of nine commonly consumed berries, namely blackberry, black goji berry, blueberry, mulberry, red Chinese bayberry, raspberry, red goji berry, strawberry and white Chinese bayberry, which may provide helpful information about the health benefits of different types of berries and offer consumers useful consumption guidance.

## 2. Materials and Methods

### 2.1. Fruit Materials

**Figure 1:**
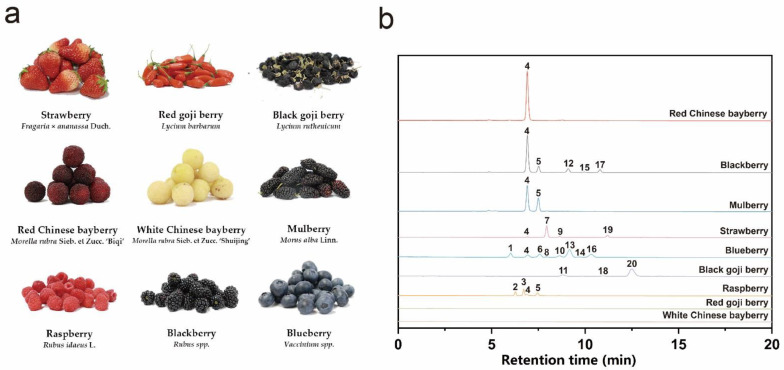
Fruit materials and berry anthocyanin profiles. (**a**) Berries studied in the present research. (**b**) UPLC chromatogram of anthocyanin profiles identified in different kinds of berry extract (λ = 520 nm). These are as follows: 1: Dp-3-*O*-hex; 2: Cy-3-*O*-sop; 3: Cy-3-*O*-2G-glucosylrutinoside; 4: Cy-3-*O*-glc; 5: Cy-3-*O*-rut; 6: Pet-3-*O*-hex; 7: Pel-3-*O*-glc; 8: Cy-3-*O*-ara; 9: Pel-3-*O*-rut; 10: Pet-3-*O*-ara and Peo-3-*O*-hex; 11: *N*,*N*′-dicaffeoylspermidine; 12: Cy-3-*O*-xyl; 13: Mv-3-*O*-gal; 14: Mv-3-*O*-glc; 15: Cy-3-*O*-(6-*O*-malonyl-β-d)-glc; 16: Mv-3-*O*-ara; 17: Cy-3-*O*-dioxalylglucoside; 18: Dp-3-*O*-rut(*trans*-*p*-coumaroyl)-5-*O*-glc; 19: Pel-3-*O*-malonylglucoside; 20: Pet-3-*O*-rut(*cis*-*p*-coumaroyl)-5-*O*-glc or Pet-3-*O*-rut(*trans*-*p*-coumaroyl)-5-*O*-glc. Abbreviations: dp: delphinidin; cy: cyanidin; pet: petunidin; pel: pelargonidin; peo: peonidin; mv: malvidin; hex: hexoside; sop: sophoroside; glc: glucoside; rut: rutinoside; ara: arabinoside; xyl: xyloside; gal: galactoside.

In total, nine kinds of berry were purchased from the local commercial market. These berries were blueberry (BLUB, *Vaccinium* spp.); black berry (BLAB, *Rubus* spp.); black goji berry (BGJB, *Lycium ruthenicum*); mulberry (MULB, *Morus alba* L.); red Chinese bayberry (RCBB, *Morella rubra* Sieb. et Zucc. ‘Biqi’); raspberry (RASB, *Rubus idaeus* L.); red goji berry (RGJB, *Lycium barbarum*); strawberry (STRB, *Fragaria × ananassa* Duch.); and white Chinese bayberry (WCBB, *Morella rubra* Sieb. et Zucc. ‘Shuijing’). Fruits without mechanical damage, diseases or pests were selected, photographed and stored at 4 °C for further experiment. Berries used in the present study were displayed in Figure 1a.

### 2.2. Extraction and Identification of Berry Anthocyanins

A total of 500 g of fresh berry pulp was accurately weighed and ground with a blender and extracted with 2 L ethanol that was acidified with formic acid (0.1%, *v*/*v*). Black goji berries were ground into powder and 50 g powder was extracted with 1 L acidified ethanol. The extract was then ultrasonically extracted with a frequency of 53 kHz for 1 h. Ice was added in the ultrasonic bath in case of temperature rising. The extract was left still overnight for sufficient extraction. The supernatant was collected after filtration. The supernatant was then concentrated to about 100 mL with a vacuum rotatory evaporator at 30 °C. The whole extraction process was conducted in shade to prevent anthocyanin breakdown.

Sep-pak C18 cartridge columns (20 cc, 5 g sorbent, Waters Corp., Milford, MA, USA) were used for the purification of anthocyanins. Briefly, after the sample was loaded, double distilled water was added to remove organic acids, sugars and other high polarity components. After that, 100% methanol was applied to elute the phenolics-rich fractions. The eluent was vacuum dried at 30 °C and kept at −18 °C for subsequent evaluation.

A structure characterization of berry anthocyanins was carried out by UPLC-Triple-TOF/MS according to our previous publication [17] with modifications. Briefly, berry extract samples were dissolved in HPLC-grade methanol with 0.1% formic acid to prepare 1 mg/mL solutions. The solutions were then filtered with 0.22 μm membrane for LC-MS analysis which was carried out on an ACQUITY UPLC/AB Triple TOF 5600^plus^ system (AB SCIEX, Framingham, MA, USA) equipped with an ESI (electrospray ionization source) system. UPLC was performed on an ACQUITY UPLC^®^ HSS T3 (1.8 μm, 2.1 × 150 mm) column. Mobile phases A and B were 1% formic acid (*v*/*v*) in water and acetonitrile, respectively. The optimized elution program was as follows: 0–5 min, 5–15% B; 5–12 min, 15–25% B; 12–20 min, 25–60% B; 20–23 min, 60–100% B; 23–24 min, 100–5% B; 24–28 min, 5% B. The monitoring UV wavelength was set at 520 nm, the flow rate was 0.3 mL/min, the temperature was 50 °C and the injection volume was 4 μL. The mass spectrometry analysis was operated in both positive and negative ion mode. The optimal parameter was as follows: the scan range of precursor ions and product ions were set as 100–1500 *m*/*z* and 50–1500 *m*/*z*, respectively. The pressure of the nebulizer gas (gas 1) and heater gas (gas 2) was set to 50 psi. The curtain gas (N_2_) pressure was 35 psi. The turbo spray temperature (TEM) was set at 600 °C (positive mode) and 550 °C (negative mode). The ion spray voltage floating (ISVF) was set at 5500 V (positive mode) and −4500 V (negative mode). The declustering potential (DP) was set as ±100 V. The collision energy (CE) was set as 40 V and −10 V, respectively. The exact mass calibration was performed automatically before each analysis by employing the automated Calibration Delivery System (CDS).

### 2.3. In Vitro Chemical Antioxidant Capacity Evaluation of Berry Extract

Four chemical antioxidant capacity evaluation assays (2,2-diphenyl-1-picrylhydrazyl (DPPH) free radical scavenging assay; 2,2′-azinobis (3-ethylbenzothiazoline-6-sulphonic acid) (ABTS) radical scavenging assay; ferric-reducing antioxidant power (FRAP) assay; and oxygen radical absorbance capacity (ORAC) assay) were applied to evaluate the antioxidant capacity of the berry extract according to our previous publication with minor modifications [18].

(1) DPPH free radical scavenging assay. A total of 2 μL of appropriate diluted berry extract was added to 198 μL DPPH solution (60 μM). After incubation in the dark for 2 h at room temperature, the absorbance at 517 nm was measured. Trolox solutions with a series of concentrations were used to plot the calibration curve and methanol was set as a blank control. The DPPH free radical scavenging activity was calculated by the following formula (equation):

DPPH radical scavenging activity (%) = [1 − (A_i_/A_0_)] × 100%

where A_i_ refers to the absorbance of samples or standards, A_0_ refers to the absorbance of the blank control. 

(2) ABTS radical scavenging assay. ABTS radical cation (ABTS^+·^) was generated by reacting ABTS solution (7 mmol/L) with potassium persulfate solution (2.6 mmol/L) (*v*:*v* = 1:1). After incubation in the dark for 12 h at room temperature, ABTS^+·^ solution was diluted with phosphate buffered saline to an absorbance of 0.70 ± 0.02 at 734 nm. A total of 10 μL of appropriately diluted berry extract was added to 200 μL ABTS^+·^ solution. After 5 min, absorbance at 734 nm was measured. Trolox solution and methanol were used as the calibration solution and blank control, respectively. The ABTS free radical scavenging activity was calculated by the following formula (equation):

ABTS radical scavenging activity (%) = [1 − (A_i_/A_0_)] × 100%

where A_i_ refers to the absorbance of samples or standards, A_0_ refers to the absorbance of the blank control. 

(3) FRAP assay. FRAP working reagent was prepared daily by mixing 300mM acetate buffer (pH = 3.6), 10 mM TPTZ solution in 40mM hydrochloric acid and 20 mM ferric chloride solution with a volume ratio of 10:1:1. A total of 10 μL of appropriate diluted berry extract was added to 90 μL FRAP working reagent. After incubation in the dark for 5 min, absorbance at 593 nm was measured. Trolox solution and methanol were used as the calibration solution and blank control, respectively.

(4) ORAC assay. The reaction was carried out in 75 mM phosphate buffer (pH = 7.4). A total of 25 μL of appropriately diluted berry extract was added into 150 μL sodium fluorescein (40 nmol/L). After incubation at 37 °C for 10 min, 25 μL AAPH solution (150 mM) was added. Then the fluorescence was recorded for 2 h with excitation and emission wavelengths of 485 nm and 535 nm, respectively. Trolox solution and phosphate buffer were used as the calibration solution and blank control, respectively. The area under the curve was calculated according to the following formula:

AUC = 2 × (f_0_ + f_1_ + f_2_ + … + f_n_) − f_n_ − f_0_

where f_n_ refers to the relative fluorescence of the nth time, f_n_ = fluorescence of the nth time/the initial fluorescence. The net AUC of the sample was calculated by subtracting the AUC of the blank control. The regression equation between net AUC and Trolox concentration was determined. 

All samples were analyzed in triplicate and all the results above were expressed as mg Trolox equivalent antioxidant capacities (TEAC)/g DW. 

### 2.4. Animals and Treatment

Six-week-old male Institute of Cancer Research (ICR) mice (25 ± 2 g) of specific pathogen-free (SPF) grade were purchased from Hangzhou Medical College and bred in the Laboratory Animal Center of Zhejiang University. All the mice were housed in a controlled environment (temperature controlled at 23 ± 3 °C, 12 h daylight cycle) with food and water ad libitum. The lab mice diet was purchased from Jiangsu Xietong Pharmaceutical Bio-engineering Co., Ltd. (Nanjing, China). After acclimation for a week, the mice were randomly divided into ten groups (n = 7/group), among which one was set as the control group and the other nine were set as treatment groups. For the treatment groups, the mice were given corresponding berry extract water solutions with a dose of 50 mg·kg^−1^ BW·d^−1^ by gavage. The control group was given equivalent distilled water. This animal experiment lasted for 14 days and the body weight and food intake of the mice were recorded weekly. At the end of the experiment, the mice were sacrificed by cervical dislocation, and the serum, liver, intestine and intestinal content was collected immediately, snap-frozen in liquid nitrogen and kept at −80 °C. All the protocols of this animal experiment were approved by the Committee on the Ethics of Animal Experiments of Zhejiang University. The permission number was ZJU20210316.

### 2.5. 16 S rDNA Amplicon Sequencing and Analysis

The total DNA of the intestinal content was extracted using a MagPure Stool DNA KF kit B (Magen, Hong Kong, China) and quantified with a Qubit Fluorometer by using the Qubit dsDNA BR Assay kit (Invitrogen, Carlsbad, CA, USA). The quality of the DNA was checked by running aliquot on 1% agarose gel. Variable regions V4 of bacterial 16S rDNA was amplified with degenerate PCR primers 515 F (5′-GTGCCAGCMGCCGCGGTAA-3′) and 806 R (5’-GGACTACHVGGGTWTCTAAT-3′). Detailed PCR cycling conditions were listed in Table S1. Libraries were qualified by the Agilent Technologies 2100 bioanalyzer. The validated libraries were used for sequencing on the Illumina HiSeq 2500 platform (BGI, Shenzhen, China) following the standard pipelines of Illumina, and generating 2 × 250 bp paired end reads. Raw reads were filtered to obtain clean data. The clean data were further classified into operational taxonomic units (OTUs) with a cutoff value of 97%. OTU representative sequences were taxonomically classified based on the RDP database (Release 16, 20160930).

### 2.6. In Vivo Antioxidant Capacity Evaluation

Appropriate amounts of liver and colonic tissue (100 mg approximately) were weighed and sufficiently homogenized with 10 volumes of cold PBS by a homogenizer (FastPrep-24^TM^ 5G, MP Biomedicals, Irvine, CA, USA). After centrifuging at 12,000 rpm for 5 min, the supernatant was gathered for further evaluation. Appropriate dilution solutions of mice serum, liver and colon homogenate were used for in vivo antioxidant capacity evaluation. The levels of total antioxidant capacity (T-AOC), total superoxide dismutase (T-SOD) activity, catalase (CAT) activity, glutathione peroxidase (GSH-PX) activity, reduced glutathione (GSH) content and malondialdehyde (MDA) content were determined with corresponding commercial kits (Nanjing Jiancheng Bioengineering Institute, Nanjing, China).

### 2.7. Statistical Analysis

Chromatographic data were processed by PeakView software (version 1.2, AB SCIEX, Toronto, ON, Canada). The statistical analysis was performed by IBM SPSS Statistics 23 software (IBM, Chicago, IL, USA). All data were expressed as mean ± SEM. A Kolmogorov-Smirnov Test (KS Test) was used to determine the sample distribution. A one-way analysis of variance (ANOVA) with Tukey’s multiple comparisons (for parametric data sets) and a Kruskal-Wallis test (for non-parametric data sets) was used for multi-group comparisons. Independent Samples Student’s *t*-test (for parametric data sets) and Mann-Whitney *U* test (for non-parametric data sets) were used for two groups comparison. *p* < 0.05 was considered statistically significant. Alpha and beta diversity were estimated by MOTHUR (v1.31.2) and QIIME (v1.8.0) at the OTU level, respectively. A sample cluster was conducted by QIIME (v1.8.0) based on UPGMA. Graphs were plotted by GraphPad Prism 8.0 (GraphPad Software, San Diego, CA, USA) and OriginPro 2021 Learning Edition (Microcal Software Inc., Northampton, MA, USA).

## 3. Results

### 3.1. Anthocyanin Profiles of Different Kinds of Berries

UPLC-Triple-TOF/MS was applied to identify the anthocyanins in different kinds of berries. UPLC chromatograms of the berries under 520 nm were merged into Figure 1b. The retention time was slightly adjusted based on Peak 4 (Cyanidin-3-*O*-glucoside). The structure characterization was assigned by comparison of the obtained mass spectrometric data with the previously published literature and the results are presented in Table 1, including the retention time, mass to charge ratio (*m*/*z*) data, molecular formula and tentative identification with specific reference. As shown in Table 1, a total of 21 substances were identified, 20 of which belong to anthocyanin. The determined anthocyanins are derivatives of the mostly mentioned six aglycones: cyanidin (*m*/*z* 287); peonidin (*m*/*z* 301); pelargonidin (*m*/*z* 271); delphinidin (*m*/*z* 303); petunidin (*m*/*z* 317) and malvidin (*m*/*z* 331). These aglycones were glycosylated with different sugar moieties in the 3-OH position. The anthocyanin profile varied in content and kind with different berry species, while there were still some similarities. No anthocyanin was detected in white Chinese bayberry and red goji berry. Cyanidin-3-*O*-glucoside (Peak 4) was distributed in most of the studied berries and the order ranked by the content from high to low was red Chinese bayberry, blackberry, mulberry. These were much higher than blueberry, raspberry and strawberry. It is also worth mentioning that cyanidin-3-*O*-glucoside was the only anthocyanin that presented in red Chinese bayberry, indicating that it may be a good resource for cyanidin-3-*O*-glucoside. Anthocyanins in blackberry, mulberry and raspberry were all cyanidin derivatives, and the main anthocyanin in strawberry and black goji berry were pelargonidin (Peak 7) and petunidin (Peak 20), respectively. Blueberry contained the most varied kinds of anthocyanins. A total of 8 anthocyanins were detected in blueberry and all the aglycones mentioned above except pelargonidin were presented in blueberry with malvidin having the highest content.

### 3.2. Antioxidant Capacity Evaluation of Different Kinds of Berries

**Figure 2:**
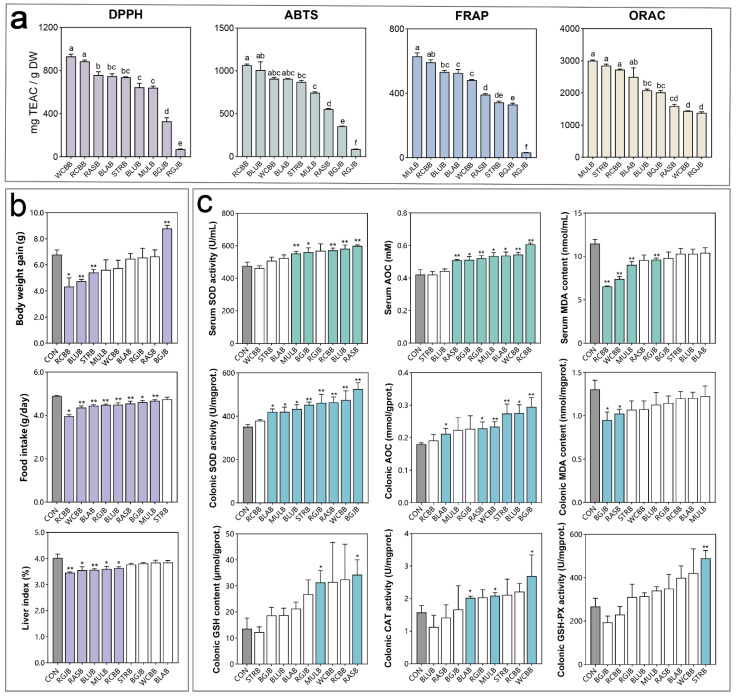
Evaluation of in vitro and in vivo antioxidant capacities of different kinds of berries. (**a**) Antioxidant capacities evaluation of different kinds of berry extract based on DPPH, ABTS, FRAP and ORAC. All samples were analyzed in triplicate. Error bars were expressed as mean ± SEM. Statistical significance was determined by one-way ANOVA with Turkey tests for multiple-group comparisons. Different letters mean significant difference between the groups (*p* < 0.05). (**b**) Influence of berry extracts on the physical condition and (**c**) antioxidant capacities of healthy objects. Error bars were expressed as mean ± SEM (n = 7/group). Statistical significance was determined by Mann-Whitney *U* test for two groups comparisons. *, compared with control group. *, *p* < 0.05; **, *p* < 0.01.

Natural antioxidants are usually multifaceted, so it is problematic to use a one-dimensional method to evaluate multifunctional food and biological antioxidants. In order to comprehensively assess the antioxidant capacities of different kinds of berries, four chemical assays based on two categories were applied. These were DPPH, ABTS, FRAP (single electron transfer (ET) reaction-based assays) and ORAC (hydrogen atom transfer (HAT) reaction-based assays). The results are displayed in Figure 2a. 

DPPH assay is often applied to assess the primary antioxidant capacity. In this assay, the antioxidant activity of the berries varied significantly between 69.63 ± 2.85 and 931.89 ± 20.82 mg TEAC/g DW. Among the berries, white Chinese bayberry and red Chinese bayberry showed the highest DPPH value with no significant difference, while red goji berry had the lowest value.

The ABTS assay evaluates the free radical scavenging ability of antioxidants. In the present assay, the antioxidant activity of berries ranged significantly from 88.10 ± 1.53 to 1065.09 ± 15.50 mg TEAC/g DW. Red Chinese bayberry, blueberry, white Chinese berry and blackberry had relatively stronger antioxidant activities with no significant difference, while red goji berry had the weakest antioxidant activity.

In the FRAP assay, [Fe(III)(TPTZ)]^3+^ is reduced into [Fe(II)(TPTZ)]^2+^ by the action of electron-donating antioxidants in an acid environment. As reflected by this assay, the FRAP values varied significantly from 32.38 ± 0.46 to 629.8 ± 21.51 mg TEAC/g DW. Mulberry and red Chinese bayberry showed the strongest antioxidant activities with no significant difference, far higher than the weakest red goji berry.

The ORAC assay is reported to be more relevant because it utilizes a biologically relevant radical source. ORAC values ranged significantly from 1376.83 ± 33.60 to 3001.27 ± 22.43 TEAC/g DW. Mulberry, strawberry, red Chinese bayberry and blackberry showed relatively higher antioxidant capacities, while those of white Chinese berry and red goji berry ranked last with no significant difference.

### 3.3. Influence of Berry Extracts on the Antioxidant Capacities of Healthy Objects

In order to investigate the effects of the berry extracts on the physical condition of healthy objects, especially the influence on the internal antioxidant capacities, we further carried out a two-week animal experiment. The results are displayed in Figure 2. As shown in Figure 2b, the intake of all the berry extracts except black goji berry alleviated the body weight gain, while only red Chinese bayberry, blueberry and strawberry reached a significant level. We recorded the food intake of each group twice a day and the results show that the food intake of the berry groups was significantly lower than that of the control group. Accordingly, we speculated that the intake of berry extract might decrease the mice appetite, and the lower body weight gain might be attributed to the lower energy intake. We next evaluated the antioxidative levels of serum, colon tissue and liver. As shown in Figure 2c, for serum, after the intake of berry extract, the levels of T-SOD and T-AOC showed an increasing trend and the level of MDA showed a decreased trend compared with the control group, and most of the berry groups reached a significant level. However, no significant differences were found in the serum GSH content and the treatment resulted in a decrease in serum CAT content (Figure S1). Similarly, for colon tissue, berry extract consumption significantly elevated the T-SOD and T-AOC levels and resulted in an increased trend in GSH content, CAT activity and GSH-PX activity, as well as a decreased trend in MDA content. Berry intake significantly decreased the liver index. Nevertheless, there was no significant difference in hepatic T-SOD level (Figure S1).

### 3.4. Influence of Berries Extracts on the Gut Microbiota of Healthy Objects

**Figure 3:**
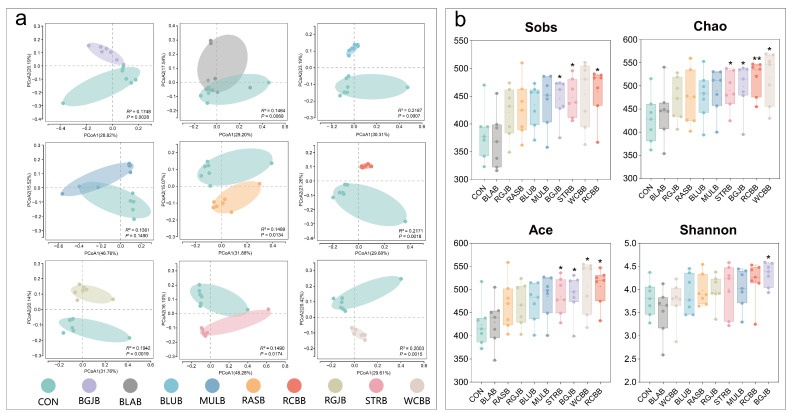
Administration of berries extracts significantly altered the gut microbiota composition. (**a**) Principal coordinate analysis (PCoA) plot based on Unweighted UniFrac matrix of the gut microbiota composition at the OTU level from different groups (n = 7/group). Pairwise comparisons using the permutational multivariate analysis of variance (PERMANOVA) test. (**b**) Alpha diversity analysis of gut bacterial richness (sobs, ace and chao indices) and diversity (Shannon index) from different mouse groups (n = 7/group), statistical significance was determined by Mann-Whitney *U* test for two groups comparisons. *, compared with control group. *, *p* < 0.05; **, *p* < 0.01.

In order to systematically assess and compare the overall composition of the gut microbiota of different berry treatments, we performed bacterial 16S rDNA amplicon sequencing on the intestinal content from all groups. A total of 830 OTUs were detected at the level of 97% similarity, and the outcome of the species–accumulation curves had reached a saturated plateau, indicating that the sequencing samples were sufficient for further data mining. Principal analysis (PCA) and Unweighted UniFrac distance matrix-based principal coordinates analysis (PCoA) were firstly conducted to visualize the differences of the bacterial communities between the control group and each berry group at OTU level. As displayed in Figure 3a and Figure S2, samples of each berry group showed a significant distinct change compared with those of the control group (PERMANOVA, *p* < 0.05), suggesting that all the berries supplementation led to drastic differences in the gut microbiota structure. In order to clearly present the relationship among ten groups, we further performed partial least squares discriminant analysis (PLS-DA). As depicted in Figure S3, there were clear clustering patterns between each group. Alpha diversity, including richness (represented by sobs, ace and chao indices) and diversity (represented by Shannon and Simpson indices) within each group was next assessed. As exhibited in Figure 3b and Figure S4, sobs, chao, ace and Shannon indices showed a similar increasing tendency while Simpson index showed a decreased tendency with berry supplementation, and the values of strawberry, black goji berry, white Chinese bayberry and red Chinese bayberry were significantly higher than that of the control group, indicating that berry intake could elevate the alpha diversity of the gut microbiota.

**Figure 4:**
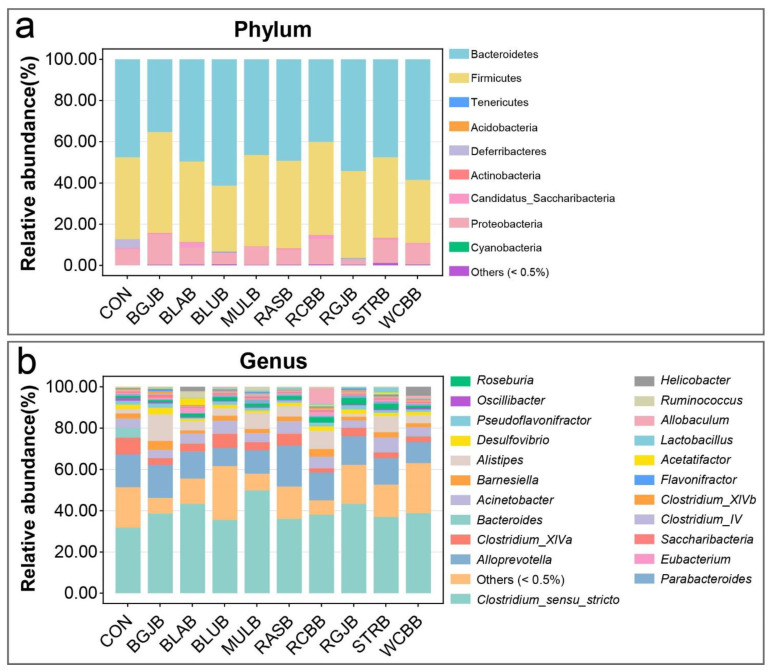
Bacterial taxonomic profiling at the (**a**) phylum level and (**b**) genus level of gut bacteria from different mouse groups (n = 7/group).

**Figure 5:**
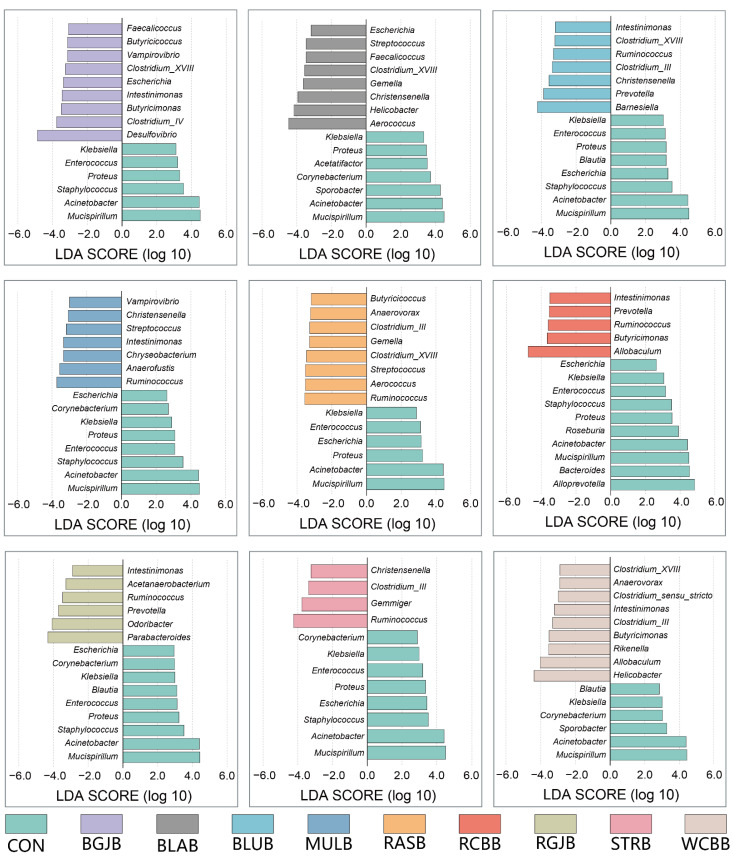
LEfSe analysis for differential abundant taxa detected between control group and each berry group. Threshold parameters were set as *p* = 0.05 for the Mann-Whitney *U* test. Linear discriminant analysis (LDA) score was >2.0.

**Figure 6:**
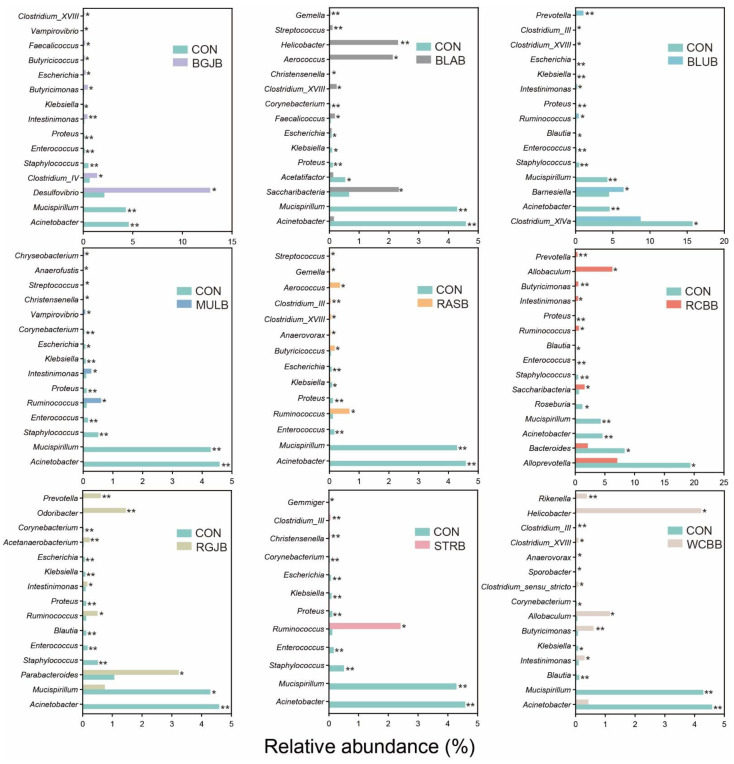
The relative abundance of top 15 genera with significant difference compared with control group in each berry group. Statistical significance was determined by Mann-Whitney *U* test for two groups comparisons. *, compared with control group. *, *p* < 0.05; **, *p* < 0.01. (n = 7/group).

**Figure 7:**
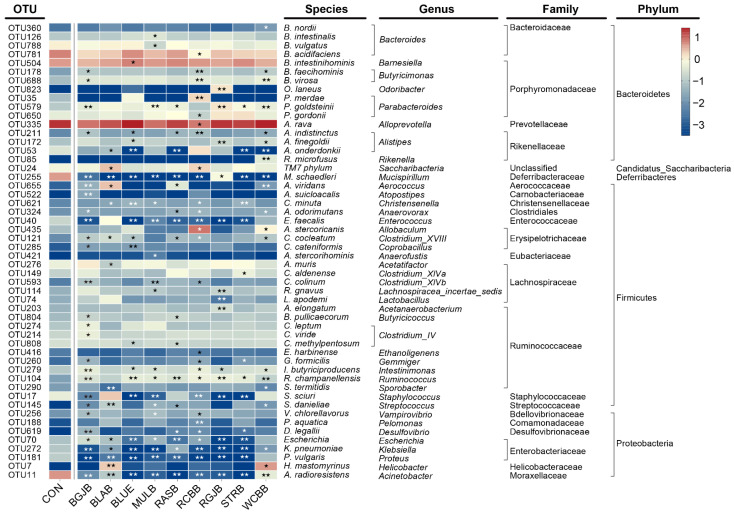
Heatmap of the bacterial species significantly altered by each berry administration. Statistical significance was determined by Mann-Whitney *U* test for two groups comparisons. *, compared with control group. *, *p* < 0.05; **, *p* < 0.01. (n = 7/group).

To assess the effect of berry administration on the overall composition of the gut microbiota, we analyzed the degree of bacterial taxonomic similarity at phylum, class, order, family and genus levels (Figure 4 and Figure S5). At phylum level, the predominant bacterial phyla were Bacteroidetes, Firmicutes and Proteobacteria (Figure 4a). The taxonomic composition distribution of the bacterial profiles based on their relative abundance at genus level was exhibited in Figure 4b. As we can see, a total of 22 genera (relative abundance > 0.5%) were annotated. At the present stage, we have a basic understanding of the overall microbial structure. However, we still lacked any information about the specific bacteria that had a significant discrimination among groups. To this end, we performed significant difference analysis and linear discriminant analysis (LDA) coupled with effect size measurements (LEfSe) for each berry group at the genus level. The top 15 genera with significant differences compared with the control group in each berry group were selected and their corresponding LDA score is shown in Figure 5. The higher the value of LDA, the greater the impact of species abundance on the difference between the control group and each berry group. A total of 42 significantly different genera were identified and the detailed comparison was summarized in Figure 6. Interestingly, the most striking discovery emerging from the data is that some genera showed a similar trend regardless of the kind of berries: *Acinetobacter*, *Clostridium_XVIII*, *Corynebacterium*, *Enterococcus*, *Escherichia*, *Intestinimonas*, *Klebsiella*, *Mucispirillum*, *Proteus*, *Ruminococcus*, *Staphylococcus*. We further performed a Spearson correlation analysis between in vivo antioxidant indices and the above genera, and the results are displayed in Figure S6. Moreover, compared with the control group, the administration of WCBB significantly enriched *Butyricimonas*, *Allobaculum*, *Helicobacter* and *Rikenella*; RGJB significantly enriched *Parabacteroides*, *Prevotella*, *Odoribacter* and *Acetanaerobacterium*; BGJB significantly enriched *Desulfovibrio*, *Clostridium_IV* and *Butyricimonas*; BLAB significantly enriched *Saccharibacteria*, *Faecalicoccus*, *Clostridium_XVIII*, *Aerococcus*, *Helicobacter*, and *Streptococcus*; BLUB significantly enriched *Prevotella* and *Barnesiella*; RASB significantly enriched *Aerococcus* and *Butyricicoccus*; and RCBB significantly enriched *Prevotella*, *Allobaculum*, *Butyricimonas* and *Saccharibacteria*. On the other hand, after RCBB administration, the relative abundance of *Alloprevotella*, *Bacteroides*, and *Roseburia* significantly decreased. BLAB decreased *Acetatifactor* and BLUB decreased *Clostridium_XIVa*. We further screened the significantly different bacteria at species level and the results are summarized in Figure 7. A total of 52 species with significant differences were identified. Red Chinese bayberry was the berry with the most kinds of significant species (27 species), while strawberry and red goji berry had the fewest kinds of significant species (14 species) compared with the control group. From Figure 7, we could also find the corresponding species of the genera that were significantly altered. For example, the alteration in the relative abundance of *Acinetobacter*, *Mucispirillum*, *Ruminococcus*, *Staphylococcus*, *Allobaculum* and *Helicobacter* were mostly explained by the reads assigned to species *A. radioresistens*, *M. schaedleri*, *R. champanellensis*, *S. sciuri*, *A. rava* and *H. mastomyrinus*, respectively.

## 4. Discussion

Previous in vitro and in vivo studies have explored the antioxidant activity and gut microbiota modulatory effects of various kinds of berries, including some of the berries in the present study. Nevertheless, there are still some limitations, especially for the latter. Most of the studies focused upon the activity of single berry phenolic compounds, overlooking that different phenolic compounds may have additive, synergistic or antagonistic effects. It should be kept in mind that the combination of the single purified nutrient or phytochemical cannot simulate the effect of the complex mixture in the plant itself. Thus, whole berry extract may be a better choice to comprehensively investigate the overall impact of berries on the gut microbiota [27,28]. Berries are more likely to be a dietary supplement for the human body to maintain health, rather than being used as therapeutic drugs for certain diseases. On the other hand, regarding berry-gut microbiota related studies, most paid attention to the effects of berries on gut microbiota that has already been disrupted by physiological disorders such as inflammatory bowel disease (IBD), obesity, diabetes, or neurodegenerative disease. Few studies investigated the effects of berries on the gut microbiota of healthy objects. Moreover, owing to the difference in experiment methods, experiment duration, experimental animal strains, materials used for bacterial DNA sequencing (intestinal content or feces), sequencing platform or databases used for species annotation (Greengene, Silva or RDP), even the results of studies investigating the gut microbiota modulatory effects of the same type of berry may be inconsistent, let alone those comparing the gut microbiota modulatory effects among different types of berries. Therefore, we evaluated and compared the antioxidant capacity and gut microbiota modulatory effect of nine common berries at the same time.

Berry fruits are especially rich in phenolic compounds, and anthocyanin was one of the most extensively studied constituents. The profile of anthocyanins varies with berry species and can be used for taxonomic purposes. Hence, we paid attention to the anthocyanin profile of the studied berries in the present experiment. We obtained phenolics-rich fractions of different berries through solid-phase extraction and identified their anthocyanin profiles by UPLC-Triple-TOF/MS. As expected, the identified anthocyanins were based on the six most common anthocyanidins, most of which were glycosylated in the 3-OH position with glucose, galactose, xylose, arabinose, rutinose or sophorose. The results of the present study were qualitatively similar yet quantitatively different from previous studies. This was not hard to comprehend as the anthocyanin content was not only subjectively related with varieties in pre- and postharvest conditions, but also objectively influenced by the extraction methods and chromatographic detection condition. There were no anthocyanins, or the anthocyanin contents were too scarce to detect in white Chinese bayberry and red goji berry. Liu et al. [29] performed a polyphenol characterization of white Chinese bayberry and indicated that the predominant polyphenols in white Chinese bayberry are proanthocyanidins, including epigallocatechin gallate, as well as flavonols, including myricitrin and quercetrin. Cyanidin-3-*O*-glucoside was also detected, but in an incomparably low level. Further, the red-orange pigment of red goji berry was mainly derived from carotenoids, but not anthocyanins [30]. Notably, cyanidin-3-*O*-glucoside was the only anthocyanin that presented in red Chinese bayberry and its content was the highest among the studied berries, indicating that it might be a good resource for cyanidin-3-*O*-glucoside.

Many in vitro and in vivo studies have elucidated the fact that berries possess various health-promoting effects that are partially attributable to their potent antioxidant capacity. Pap et al. [8] comprehensively narrated the recent progress of berry antioxidant activities in their review. We evaluated and compared the antioxidant capacities of the nine berries both in vitro and in vivo. All four chemical assays have been widely used to evaluate the antioxidants in natural product systems [31]. To sum up, the results of the above assays were different overall. On the one hand, different berries exhibited different antioxidant capacities in the same assay, and the differences were supposed to be attributed to the differences in their chemical compositions, especially phenolic compounds. On the other hand, the same kind of berry could have different antioxidant capacities in different evaluation methods. For example, white Chinese bayberry showed relatively high antioxidant activity in DPPH, ABTS and FRAP assays, while it showed low activities in ORAC assay. Raspberry showed strong antioxidant activity in DPPH assay, while that in ABTS, FRAP and ORAC was weak. Strawberry showed high activities in ORAC assay, while it showed moderate activity in the other three assays. Mulberry showed very strong activity in FRAP and ORAC assays, much higher than that in DPPH and ABTS. Blueberry presented high activity in ABTS and FRAP assays, while it was moderate in DPPH and ORAC assays. However, some berries still showed similar tendencies in all assays. For all assays, red Chinese bayberry showed potent antioxidant activities, blackberry showed moderate activities, while black goji berry and red goji berry showed fairly weak activities. Therefore, it is necessary to perform antioxidant capacity evaluation assays with different chemical principles. It is also important to tell the applied evaluation assay when performing antioxidant capacity comparisons or it is hard to tell which berry has a stronger antioxidant capacity.

The GI tract is directly exposed to nutrients, toxic food contaminants, therapeutic drugs, and intestinal bacteria. The collapse of the oxidative stress defense system in the GI tract is closely related with intestinal pathologies such as IBD, thus, maintaining the equilibrium of the redox state of the GI tract is not only crucial for its functionality, but also for the entire organism. GSH is one of the main non-enzymatic antioxidants in the gut. The major GSH-dependent enzymatic antioxidants include SOD, GSH-PX and CAT, and they all contribute to the antioxidant defense against gut oxidative stress [32]. While MDA is a product of free radical damage to polyunsaturated membrane lipids, an important marker of oxidative stress. 

We measured and compared the above non-enzymatic and enzymatic antioxidant indicators in healthy mice serum and colon tissues after the administration of different berry extracts for two weeks. Our results show that most berry administration increased the antioxidant markers (T-AOC, GSH, T-SOD, CAT, GSH-PX,) and decreased the oxidative stress marker (MDA) in serum and colon tissues either significantly or in a minor way, and the targets of each berry were different. In particular, red goji berry and black goji berry, which showed weak antioxidant capacities in chemical assays, showed moderately outstanding antioxidant activities in vivo. This interesting phenomenon implied that berry phenolics and their metabolites jointly played a role in the in vivo antioxidant activity. Confusingly, most berry administration led to a decrease in serum CAT activity, which is inconsistent with previous studies [8]. The underlying mechanism required further investigation. Conclusively, most of the nine berries could elevate the internal antioxidant level of healthy organisms through the improvement of oxidative defense system and the prevention of oxidative stress.

Our GI tract is colonized by millions of microorganisms, namely gut microbiota, which plays a pivotal role in maintaining the intestinal homeostasis and physiological health. A growing quantity of evidence has suggested that gut microbiota dysbiosis is involved in various diseases, thus it is vital to keep a balanced gut microbiota. Prebiotics are substrates that are selectively utilized by host microorganisms and confer a health benefit. Although current prebiotics are predominantly carbohydrate-based, substances such as polyphenols and fatty acids also exert prebiotic effects, including defense against pathogens, immune modulation, mineral absorption, bowel function, metabolic effects and satiety [33]. Berries are rich in phenolics and are proved to have prebiotic-like effects. A huge quantity of berry phenolics reach the gastrointestinal tract and are metabolized into smaller molecules by the gut microbiota. The phenolics and their metabolites, such as simple phenolic acids and phenolic acids-SCFAs conjugates [15,34] together modulate the microbial community. Laura et al. [3] recapitulated the potential modulatory effect of polyphenols from a wide range of berries on the gut microbiota and gut health in their review. According to the review, the most studied berry was blueberry, while there are still many other berries requiring deeper investigation. Berries could positively modulate the gut microbiota through promoting the growth of beneficial bacteria (*Bifidobacterium*, *Lactobacillus*, *Akkermansia*, etc.) and inhibiting the growth of potentially pathogenic bacteria (*Pseudomonas*, *Salmonella*, *Staphylococcus* or *Bacillus*, etc.). Different berry phenolics affect the bacteria selectively, and the bacteria show different sensitivity towards berry phenolics. [3] This prompted us to investigate and compare the gut microbiota modulatory effect of different types of berries. To the best of our knowledge, our study was the first time that a comparison of the gut microbiota regulatory effect of so many berries on healthy organisms has been explored. Meanwhile, the gut microbiota modulatory effect of some presently used berries has not been reported before, such as red Chinese bayberry and white Chinese bayberry. The results of PCA, PCoA and PLS-DA as a whole showed that the administration of all nine types of berries led to a dramatic shift on the gut microbiota composition. Afterwards, we measured the alpha diversity of each group. As indicated by the indices, most berry administration enhanced the richness and diversity of the gut microbiota, either significantly or in a minor way. Red Chinese bayberry, white Chinese bayberry, strawberry, black goji berry and mulberry exhibited relatively higher promoting effects. Generally, a high taxa diversity, high microbial gene richness and stable microbiome are core characteristics of a healthy microbiota [35]. A species-rich gut ecosystem is more robust against environment influences, while lower bacterial diversity has been observed to be related to various diseases [12].

We further explored the detailed changes of gut microbiota after berry administration at the phylum, family, genus and species level. Overall, the impacts of different berries on the gut microbiota were virtually the same with minor differences. Remarkably, the growth of *Acinetobacter* and *Mucispirillum* were drastically suppressed by all the berries. *Acinetobacter* [36] is a complex genus with more than 50 species, some of which are multiresistant pathogens and able to cause infection. *Mucispirillum* spp. [37,38] (e.g., *Mucispirillum schaedleri*) are linked with IBD, high fat diet, stress and a variety of other diseases. Besides, most berries administration (more than five types) significantly decreased the proportions of detrimental or opportunistic pathogens, namely *Corynebacterium*, *Proteus* [39], *Enterococcus* [40], *Klebsiella* [41], *Staphylococcus* and *Escherichia*, most of which are triggers of infections or associated with diseases such as IBD. Moreover, most berries also boosted the growth of some potentially beneficial bacteria. *Ruminococcus* [42] is an important gut microbial mutualist which serves to degrade a diversity of plant polysaccharide into a variety of nutrients for the host; *Intestinimonas* [43] could produce butyrate from both sugars and amino acids; and *Clostridium_XVIII* [44] was positively correlated with SCFAs production. As revealed by a Spearson correlation analysis, most of the potentially pathogenic bacteria were significantly negatively correlated with antioxidant indices (especially T-SOD and colonic T-AOC) and positive correlated with oxidative stress marker (MDA), indicating that the in vivo antioxidant capacity of berries may be partially related to the modulation of gut microbiota. Interestingly, some bacteria could only be modulated by certain berries. Most of the positively regulated bacteria were SCFAs-producing bacteria as well as possible health promoting bacteria, including *Butyricimonas* [45], (enriched by WCBB, BGJB and RCBB); *Allobaculum* [46] (enriched by WCBB and RCBB); *Parabacteroides* [47] (enriched by RGJB); *Prevotella* [48] (enriched by RGJB, BLUB and RCBB); *Odoribacter* [49] (enriched by RGJB); *Clostridium_IV* [50] (enriched by BGJB); *Saccharibacteria* [44] (enriched by BLAB and RCBB); *Faecalicoccus* [51] (enriched by BLAB); *Aerococcus* [52] (enriched by BLAB and RASB); *Barnesiella* [53] (promoted by BLUB); *Butyricicoccus* [49] (enriched by RASB); and *Rikenella* [54] (enriched by WCBB). The negatively regulated bacteria were generally associated with disease, including *Alloprevotella* [55] (suppressed by RCBB) and *Acetatifactor* [56] (suppressed by BLAB). Nevertheless, some berries also enriched bacteria which are commonly considered harmful, for instance, *Helicobacter* [57] (enriched by WCBB and BLAB); *Desulfovibrio* [58] (enriched by BGJB); and *Streptococcus* [59] (enriched by BLAB). These bacteria are often enriched in unhealthy physiological conditions such as gastrointestinal inflammation, anxiety and depression. Unexpectedly, the growth of some SCFAs-producing bacteria was also inhibited, including *Blautia* (suppressed by BLUB, RCBB, RGJB and WCBB); *Bacteroides*, *Roseburia* [57] (suppressed by RCBB); and *Clostridium_XIVa* [50] (suppressed by BLUB). However, we should bear in mind that interactions among microorganisms are highly complex, and the function of certain bacteria can be double-edged. In other words, they benefit the host in some settings and harm the host in other circumstances [60]. SCFAs play crucial roles in the interplay between diet, microbiota and health [50]. Studies have shown that SCFAs could prevent body weight gain by increasing energy expenditure and appetite control [61]. Previous studies have elucidated that some berries could increase SCFAs productions through the modulation of gut microbiota. Notably, our results found that the administration of most berries alleviated body weight gain and suppressed food intake. Together with the enrichment of SCFAs-producing bacteria revealed by our results and previous studies, we speculated that the intake of berry extracts elevated colonic SCFAs levels through the modulation of gut microbiota. They then controlled the appetite, eventually leading to prevention of body weight gain, but the specific mechanisms of this require further investigation. 

Accordingly, our results preliminarily suggested that the beneficial effect of berries might be partially related to their modulation on the gut microbiota. Nevertheless, there were still some limitations in our research. Firstly, it is hard to tell which berry was the winner in the present ‘competition’. Each berry has its characteristic benefits embodied in different target antioxidant enzymes and target gut bacteria. It is suggested that people should consume diverse kinds of berries daily as they may have compensatory effects. Moreover, although it has been indicated that certain bacteria are involved in modulating the host redox status and improving the antioxidant system [62], the specific linkage between berry antioxidant effect and gut microbiota in the present study remains unclear. It would be of interest to investigate the underlying mechanism from the aspect of gut microbiota.

## 5. Conclusions

In the present study, we identified the anthocyanin profiles, evaluated and compared the antioxidant capacities and gut microbiota modulatory effects of nine common berries. The identified anthocyanins were based on the six most common anthocyanidins. The in vitro antioxidant capacities of berries varied with different chemical assays. Most of the nine berries could elevate the internal antioxidant activity. All the berries administration exerted a substantial effect on the gut microbiota composition. The modulatory effects of the berries were the same in terms of the essentials but different in minor areas, indicated by the enrichment of beneficial SCFAs-producing bacteria and the inhibition of potentially harmful bacteria. Our study shed light on the gut microbiota modulatory effect of different berries and may offer consumers useful consumption guidance.

## Figures and Tables

**Table 1:** Identification of anthocyanins in different kinds of berries by UPLC-Triple-TOF/MS.

Peak No.	Retention Time/Min	MS (*m*/*z*)	MS^2^ Ions (Relative Abundance) (*m*/*z*)	Molecular Formula	Tentative Identification	References
1	6.0576	465.1039; [M]^+^	303.0496 (100)	C_21_H_21_O_12_	Delphinidin hexoside (Delphinidin-3-*O*-galactoside or delphinidin-3-*O*-glucoside)	[19,20]
2	6.3155	611.162; [M]^+^	287.0556 (100), 611.1637 (13.08)	C_27_H_31_O_16_	Cyanidin-3-*O*-sophoroside	[21]
3	6.7586	757.2215; [M]^+^	287.0552 (100), 757.2215 (38.43)	C_33_H_41_O_20_	Cyanidin-3-*O*-2G-glucosylrutinoside	[21]
4	6.9068	449.109; [M]^+^	287.0558 (100)	C_21_H_20_O_11_	Cyanidin-3-*O*-glucoside	[22]
5	7.5025	595.1671; [M]^+^	287.0557 (100), 595.1650 (11.67)	C_27_H_30_O_15_	Cyanidin-3-*O*-rutinoside	[21,23]
6	7.5919	479.1196; [M]^+^	317.0665 (100)	C_22_H_23_O_12_	Petunidin hexoside (Petunidin-3-*O*-galactoside or Petunidin-3-*O*-glucoside)	[19,20]
7	7.9218	433.1144; [M]^+^	271.0604 (100)	C_21_H_21_O_10_	Pelargonidin-3-*O*-glucoside	[23]
8	7.9453	419.0982; [M]^+^	287.0551 (100)	C_20_H_19_O_10_	Cyanidin-3-*O*-arabinoside	[19]
9	8.6335	579.1721; [M]^+^	271.0616 (100)	C_27_H_31_O_14_	Pelargonidin-3-*O*-rutinoside	[23]
10	8.6412	449.1088; [M]^+^	317.0662 (100)	C_21_H_21_O_11_	Petunidin-3-*O*-arabinoside	[24]
		463.1247; [M]^+^	301.0707 (100)	C_22_H_23_O_11_	Peonidin hexoside (Peonidin-3-*O*-galactoside or peonidin-3-*O*-glucoside)	[20,21,25]
11	8.7697	468.2126; [M-H]^−^	135.0457 (62.47), 289.1558 (19.2), 306.1827 (30.77), 332.1622 (100), 468.2156 (70.9)	C_25_H_31_N_3_O_6_	*N*,*N*′-dicaffeoylspermidine	[25]
12	9.0895	419.0983; [M]^+^	287.0559 (100)	C_20_H_18_O_10_	Cyanidin-3-*O*-xyloside	[23]
13	9.18	493.1349; [M]^+^	331.0823 (100)	C_23_H_25_O_12_	Malvidin-3-*O*-galactoside	[19,24]
14	9.7379	493.1352; [M]^+^	331.0824 (100)	C_23_H_25_O_12_	Malvidin-3-*O*-glucoside	[19,24]
15	9.9434	535.1094; [M]^+^	287.0547 (100)	C_24_H_22_O_14_	Cyanidin-3-*O*-(6-*O*-malonyl-β-d)-glucoside	[23]
16	10.2993	463.1243; [M]^+^	331.0809 (100)	C_22_H_23_O_11_	Malvidin-3-*O*-arabinoside	[24]
17	10.7913	593.1518; [M]^+^	287.0553 (100)	C_25_H_20_O_17_	Cyanidin-3-*O*-dioxalylglucoside	[23]
18	10.9592	919.2509; [M]^+^	303.0512 (100), 465.105 (12.72), 757.2022 (25.8), 919.2569 (60.23)	C_42_H_47_O_23_	Delphinidin-3-*O*-rutinoside(*trans*-*p*-coumaroyl)-5-*O*-glucoside	[26]
19	11.1733	519.1152; [M]^+^	271.0609 (100)	C_24_H_23_O_13_	Pelargonidin-3-*O*-malonylglucoside	[23]
20	12.4938	933.2703; [M]^+^	317.0668 (100), 479.1206 (33.98), 711.2168 (57.19), 933.2718 (56.51)	C_43_H_48_O_23_	Petunidin-3-*O*-rutinoside(*cis*-*p*-coumaroyl)-5-*O*-glucoside or petunidin-3-*O*-rutinoside(*trans*-*p*-coumaroyl)-5-*O*-glucoside	[25]

## Data Availability

Not applicable.

## Supplementary Materials

The following supporting information can be downloaded at: https://www.mdpi.com/article/10.3390/antiox11051020/s1, Figure S1: Influence of berry extracts on the antioxidant capacities of healthy objects. Figure S2: Principal component analysis (PCA) plot of the gut microbiota composition at the operational taxonomic unit (OTU) level from different groups. Figure S3: Partial least squares discriminant analysis (PLS-DA) of the gut microbiota composition at the operational taxonomic unit (OTU) level from different groups. Figure S4: Alpha diversity analysis of gut bacterial diversity (Simpson index) from different mouse groups. Figure S5: Bacterial taxonomic profiling of gut bacteria from different mouse groups. Figure S6: Spearman correlation analysis between gut microbiota and in vivo antioxidant indices. Table S1: PCR conditions.
