# Supplementary material for: Evaluation of Antioxidant Capacity and Gut Microbiota Modulatory Effects of Different Kinds of Berries
Source: Antioxidants (Basel). 2022 May 22;11(5):1020. doi: 10.3390/antiox11051020 (PMC9137550; doi:10.3390/antiox11051020)
Supplement: Supplementary file 1 [file antioxidants-11-01020-s001.zip › antioxidants-1727625-supplementary.pdf]

Table S1. PCR conditions

Both forward and reverse primers were tagged with Illumina adapter, pad and linker sequences. PCR enrichment was performed in a 50 μL reaction containing 30ng template, fusion PCR primer and PCR master mix. PCR cycling conditions were as follows:

Temperature	Time	Number of cycles
95°C	3 minutes	/
95°C	45 seconds	
56°C	45 seconds	30 cycles
72°C	45 seconds	
72°C	10 minutes	/

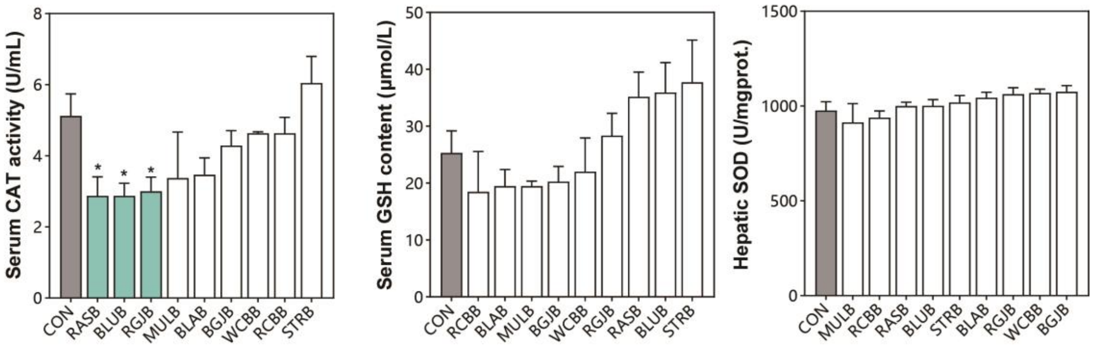

Figure S1. Influence of berry extracts on the antioxidant capacities of healthy objects. Error bars were expressed as mean ± SEM (n = 7/group). Statistical significance was determined by Mann-Whitney *U* test for two groups comparisons. *, compared with control group. *, $P < 0.05$; **, $P < 0.01$.

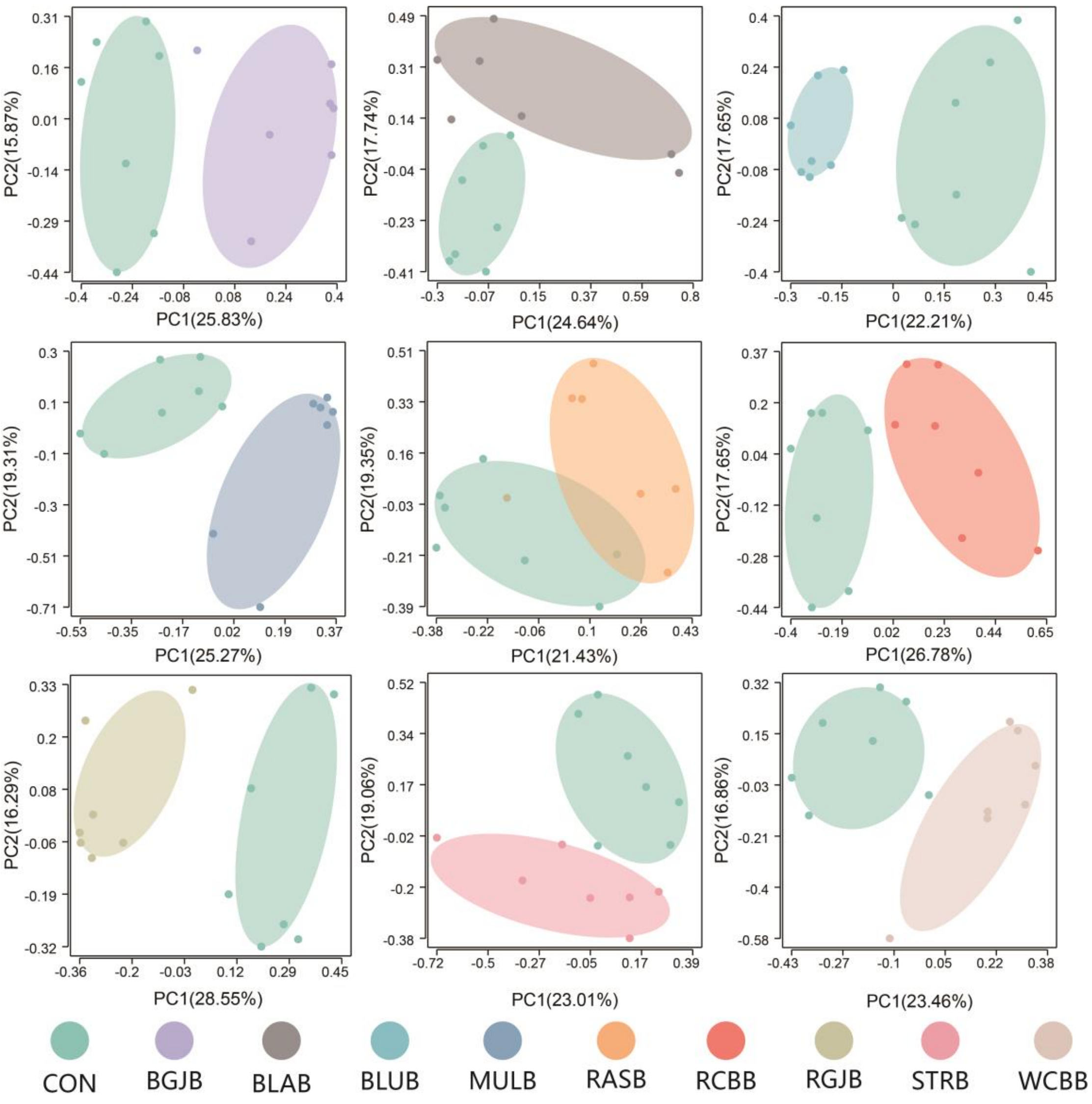

Figure S2. Principal component analysis (PCA) plot of the gut microbiota composition at the operational taxonomic unit (OTU) level from different groups (n = 7/group).

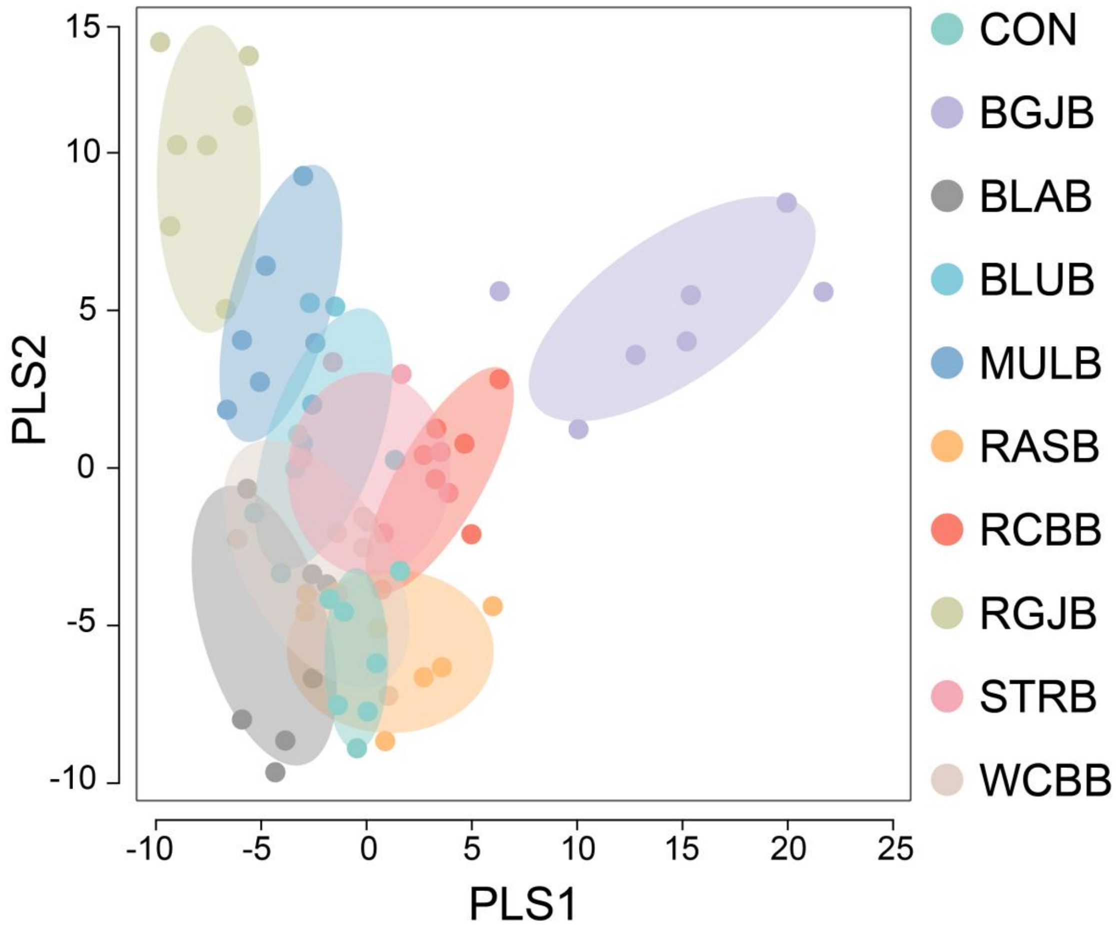

Figure S3: Partial least squares discriminant analysis (PLS-DA) of the gut microbiota composition at the operational taxonomic unit (OTU) level from different groups (n = 7/group).

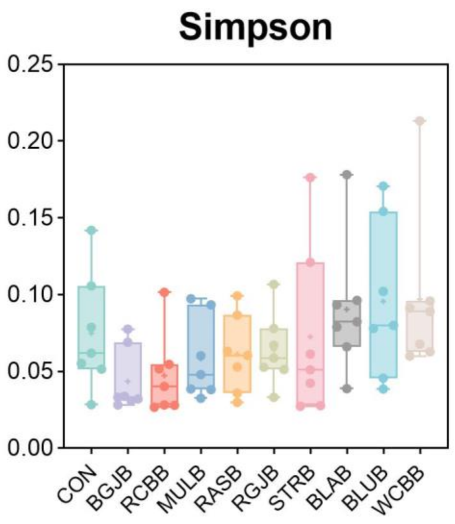

Figure S4: Alpha diversity analysis of gut bacterial diversity (Simpson index) from different mouse groups (n = 7/group).

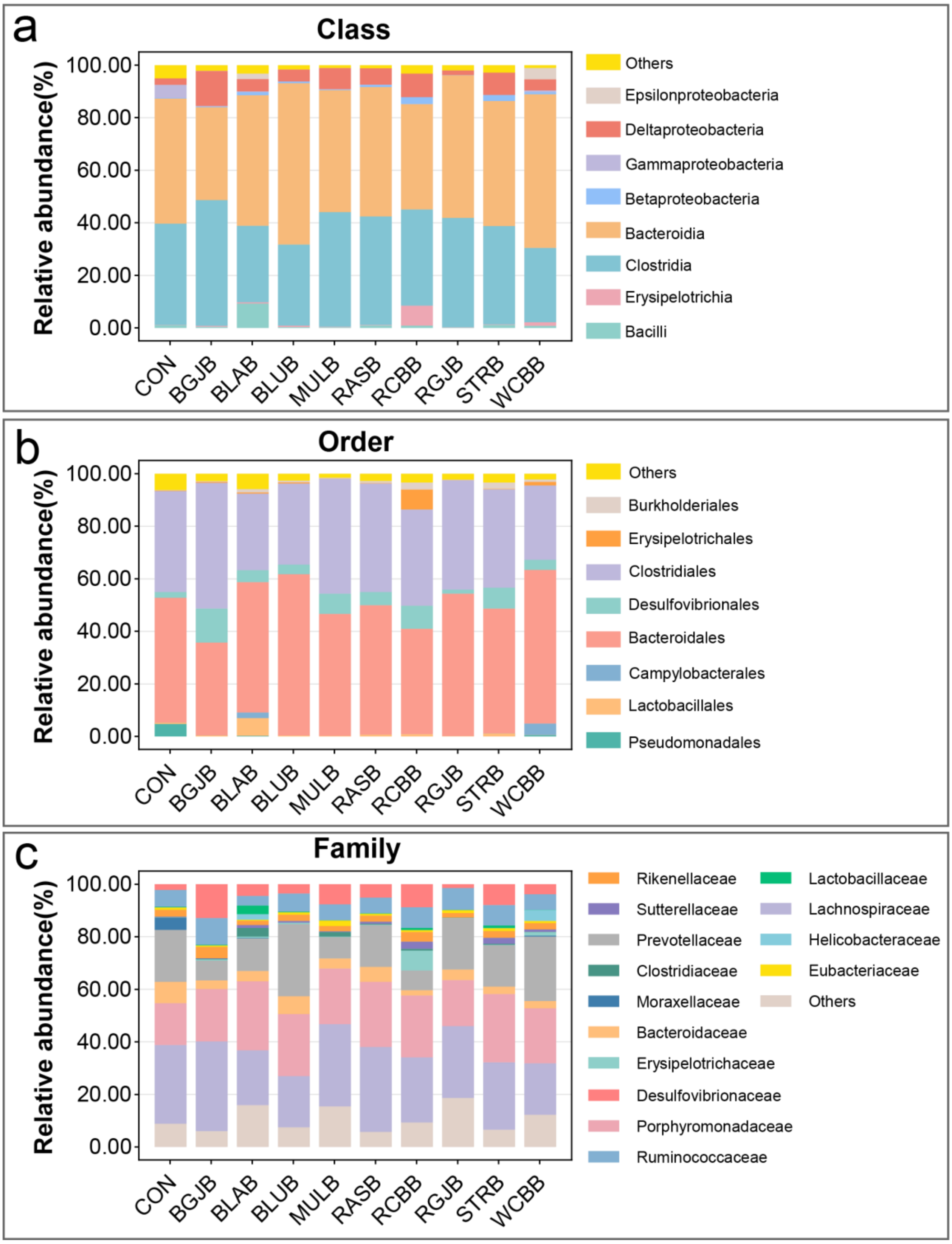

Figure S5. Bacterial taxonomic profiling at the (a) class level, (b) order level and (c) family level of gut bacteria from different mouse groups (n = 7/group).

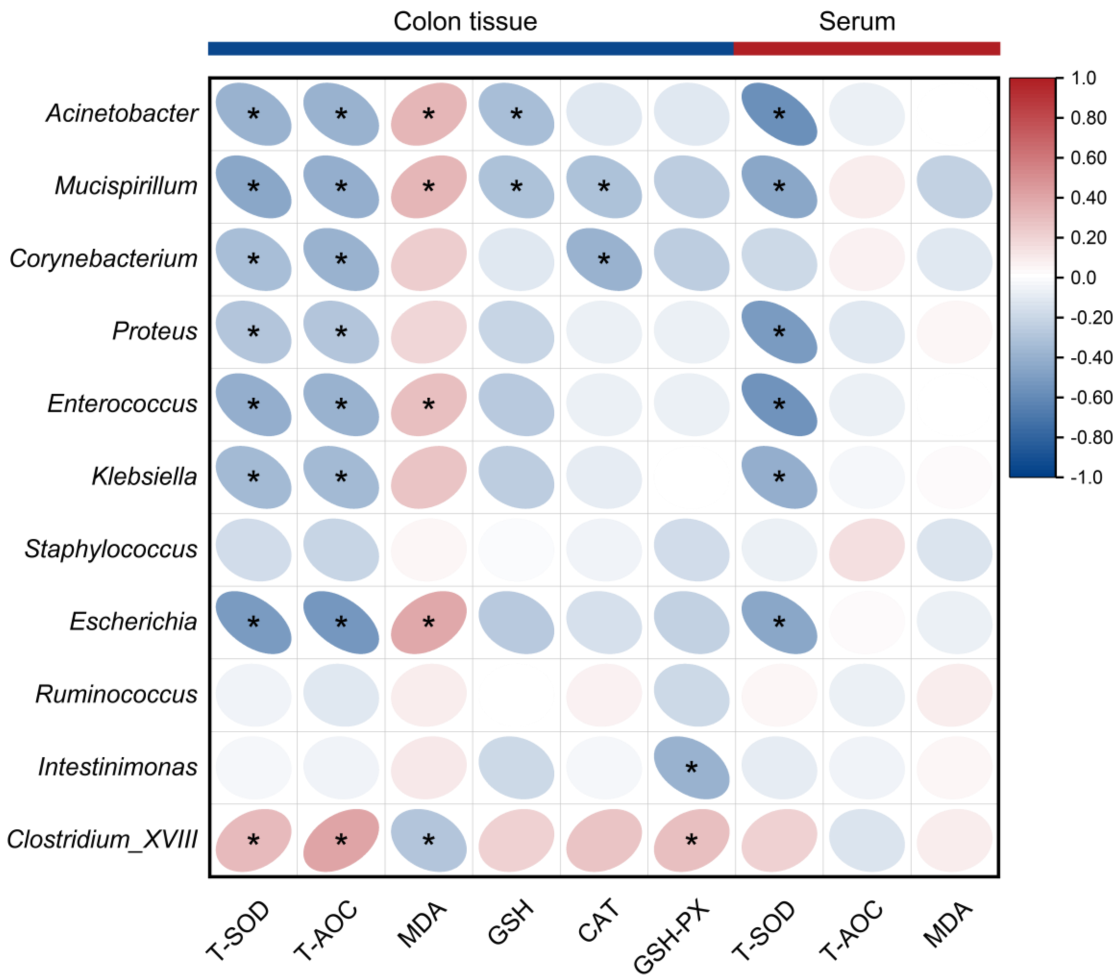

Figure S6: Spearman correlation analysis between gut microbiota and *in vivo* antioxidant indices. The red color denotes a positive correlation, while blue color denotes a negative correlation. The intensity of the color is proportional to the strength of Spearman correlation. *, $P < 0.05$.
